# Supplementary material for: TBOE maturity assessment model for standard digitalization: An empirical analysis using AHP and Delphi method
Source: PLoS One. 2026 Mar 25;21(3):e0343406. doi: 10.1371/journal.pone.0343406 (PMC13016301; doi:10.1371/journal.pone.0343406)
Supplement: S1 File — (DOCX) [file pone.0343406.s001.docx]

**S1 File. AHP expert consultation scoring table**

I. Description of the indicator system

This project utilizes the Analytic Hierarchy Process (AHP) to develop a decision-support model for standard digital transformation (SGSD-TBOE) in the power industry. The framework comprises four primary indicators: Technical Factors (T), Business factors (B), Organizational Factors (O), and Environmental Factors (E), supported by 13 secondary indicators including core technologies, grid business scenario integration, organizational leadership, and international development environment, along with corresponding value criteria. The indicator system structure is detailed in the table below.

| First-level indicators | Second-level indicators | Measurement index |
| --- | --- | --- |
| Technical factors (T) | T1:Core key technologies | Technology Readiness Level (TRL) |
|  | T2:New digital infrastructure | Number of domains covered by the standard data base |
|  | T3:Data security protection | Standard data security capability maturity |
| Business factors (B) | B1:Convergence of business scenarios | Integration degree between digital standards and traditional business scenarios of electric power |
|  | B2:Integration of new technology systems | Integration of digital standards with new power systems |
|  | B3:Integration of industrial engineering | Integration degree of electric power digital standards and major industrial projects |
| Organizational factors (O) | O1:Organization and management | Construction of the organization management system for the digitalization of standards |
|  | O2:Policy construction | Construction of supporting systems for the digitalization of standards |
|  | O3:Talent cultivation | The construction of personnel training mechanism for the digitalization of standards |
|  | O4:Funding | Proportion of R&D expenditure |
| Environmental factors (E) | E1:International development environment | Participation in international standardization activities |
|  | E2:Domestic policy environment | Support degree of national policies and regulations |
|  | E3:Industry market environment | Construction degree of digitalization of standard in the electric power industry |

II. Instructions for filling in the form

Use the 1-9 scale method to evaluate the relative importance of each indicator according to the scale meanings in the table below. For horizontal coordinates compared to vertical coordinates: 1 indicates equal importance, 3 for slightly more important, 5 for significantly more important, 7 for strongly important, 9 for extremely important, and 2-8 for intermediate levels. Conversely, use their reciprocals for lower importance levels.

| scale | meaning |
| --- | --- |
| 1 | The two factors are equally important |
| 3 | Of the two factors, the former is slightly more important than the latter |
| 5 | Of the two factors, the former is significantly more important than the latter |
| 7 | Of the two factors, the former is more important than the latter |
| 9 | Of the two factors, the former is far more important than the latter |
| 2,4,6,8 | Indicates that the values are between the above adjacent values |
| count backwards | If the ratio of importance between factor i and factor j is bij,  Then the ratio of the importance of factor j to factor i is bji=1 / bij |

III. First-level indicator scoring table (horizontal coordinate/vertical coordinate)

|  | Technical factors (T) | Business factors (B) | Organizational factors (O) | Environmental factor（E） |
| --- | --- | --- | --- | --- |
| Technical factors (T) | 1 | (Not required) | (Not required) | (Not required) |
| Business factors (B) |  | 1 | (Not required) | (Not required) |
| Organizational factors (O) |  |  | 1 | (Not required) |
| Environmental factor （E） |  |  |  | 1 |

IV. Secondary indicator scoring table (horizontal coordinate/vertical coordinate)

1. Technical factors (T)

|  | Core Key Technologies (T1) | New Digital Infrastructure (T2) | Data Security Protection (T3) |
| --- | --- | --- | --- |
| Core Key Technologies (T1) | 1 | (Not required) | (Not required) |
| New Digital Infrastructure (T2) |  | 1 | (Not required) |
| Data Security Protection (T3) |  |  | 1 |

2.Business factors (B)

|  | Convergence of business scenarios (B1) | Integration of new technology systems(B2) | Integration of industrial engineering(B3) |
| --- | --- | --- | --- |
| Convergence of business scenarios (B1) | 1 | (Not required) | (Not required) |
| Integration of new technology systems (B2) |  | 1 | (Not required) |
| Integration of industrial engineering (B3) |  |  | 1 |

3. Organizational factors (O)

|  | Organization and management (O1) | Policy construction (O2) | Talent cultivation(O3) | Funding (O4) |
| --- | --- | --- | --- | --- |
| Organization and management (O1) | 1 | (Not required) | (Not required) | (Not required) |
| Policy construction (O2) |  | 1 | (Not required) | (Not required) |
| Talent cultivation(O3) |  |  | 1 | (Not required) |
| Funding(O4) |  |  |  | 1 |

4. Environmental factor（E）

|  | International Development Environment (E1) | Domestic policy environment (E2) | Industry market environment (E3) |
| --- | --- | --- | --- |
| International Development Environment (E1) | 1 | (Not required) | (Not required) |
| Domestic policy environment (E2) |  | 1 | (Not required) |
| Industry market environment (E3) |  |  | 1 |

V. Basic information of experts (Please check the box within □)

| Your age | □ Under 40 □ 40-50 □ 50-60 □ Over 60 | | |
| --- | --- | --- | --- |
| Workplace |  | Professional title or position |  |
