# Supplementary material for: TBOE maturity assessment model for standard digitalization: An empirical analysis using AHP and Delphi method
Source: PLoS One. 2026 Mar 25;21(3):e0343406. doi: 10.1371/journal.pone.0343406 (PMC13016301; doi:10.1371/journal.pone.0343406)
Supplement: Attachment — Submitted filename: Comments-PONE-D-24-6044_R1.pdf [file pone.0343406.s005.pdf]

Comments on the paper**:**

TBOE Maturity Assessment Model for Standard Digitalization: An Empirical Analysis Using AHP and Delphi Method

1. The study gives strong practical value, especially when it is applied in the power industry, which has rich data and strict standard requirements. However, from an academic view, the level of innovation is mostly in its application and system integration**,** not in deep theoretical development. The authors should explain more clearly how the TBOE framework is different from the traditional TOE or DTF models, and show what new ideas the “Business” factor brings to the theory.

2. In terms of methodology, the consistency tests (CR) in the AHP are all within the acceptable range ($CR < 0.1$), showing that the model is mathematically reliable. Still, there are some technical points that should be improved:

   - When combining expert judgments, it is better to use the **geometric mean** instead of the arithmetic mean to match the scale of AHP.
   - The process of normalizing the indicators should be explained more clearly, especially when using different types of scales (such as 0/1, TRL levels, or count-based data).
   - The thresholds for maturity levels (0.3 and 0.8) seem a bit subjective; they should be supported with stronger arguments or real evidence.
   - The paper has not tested how stable the results are when the weights or input data change.

Overall, the authors need to describe more clearly how they calculate the weights, normalize the data, and justify the chosen thresholds. Adding sensitivity or robustness analysis would make the results more convincing and scientifically sound.

In short, the paper is well-prepared and has clear practical contributions, with real-world applications. However, to meet the publication standard of PLOS ONE*, the authors should improve the technical parts, especially in data normalization and stability testing of the model.*
