# Supplementary material for: TBOE maturity assessment model for standard digitalization: An empirical analysis using AHP and Delphi method
Source: PLoS One. 2026 Mar 25;21(3):e0343406. doi: 10.1371/journal.pone.0343406 (PMC13016301; doi:10.1371/journal.pone.0343406)
Supplement: Attachment — Submitted filename: Reviewer_Comments_to_Authors.docx [file pone.0343406.s002.docx]

Dear Authors,

Thank you for the opportunity to review your manuscript, which proposes the TBOE Maturity Assessment Model for evaluating the digitalization of standards. The paper addresses a timely and practically relevant topic—particularly as digital transformation efforts expand across industries. Your empirical focus on the electric power sector and the application of AHP and Delphi methods are commendable efforts.

However, in its current form, the manuscript falls short of the publication criteria for this journal. I therefore recommend rejection at this time. That said, I believe this work has the potential to be resubmitted elsewhere after careful revision. I hope the following detailed comments will assist you in improving the manuscript for possible submission to another journal.

**1. Theoretical Justification and Model Positioning**
While the extension from TOE to TBOE is logical, the manuscript lacks a strong theoretical rationale for adding the "Business" component. Please clarify what conceptual gaps in the existing TOE or DTF frameworks your model fills. Including a comparative discussion of related maturity models or digital transformation theories could significantly improve the paper’s contribution.

**2. Generalizability and Broader Relevance**
Currently, the model is validated only within the Chinese electric power industry. While appropriate as a starting point, readers would benefit from discussion about how the model could apply across other sectors or geographies. Addressing generalizability would improve both scholarly relevance and practical utility.

**3. Methodological Transparency**
The description of the expert survey process needs significant elaboration. For example:
- How were the 84 experts selected?
- What were their backgrounds, affiliations, and expertise levels?
- What steps were taken to minimize bias in the Delphi process?

Including this information would help readers assess the robustness of the model-building process.

**4. Validation Depth**
The model relies heavily on AHP-derived consistency ratios (CR) as the primary form of validation. Consider adding additional checks, such as sensitivity analysis, to better establish confidence in the results. Even a limited robustness test could help improve the perceived reliability of your framework.

**5. Language and Writing Quality**
There are multiple instances of unclear or overly abstract phrasing that reduce readability. For example, the phrase “digital standard is the linchpin of digital China” may not be meaningful to readers outside the specific policy context. A thorough professional English review is strongly recommended to improve the tone, clarity, and precision of the text.

**6. Abstract and Structure**
The abstract is too lengthy and includes excessive methodological detail. Try to more concisely highlight the purpose, approach, and key findings of your study.

**7. Figures and Tables**
Some visuals can be improved:
- Figure 1 lacks clear definitions of directional arrows like “drive” and “push.”
- Figure 2 is dense and could be split into parts for easier interpretation.
- Table 4 is well-organized—consider visualizing it using bar charts to enhance accessibility.

**8. References**
Make sure government and policy-related references are properly formatted and accessible to an international audience. When citing Chinese-language materials, translated titles and English summaries would be helpful.

**Final Note:**
While I am recommending rejection for this journal, I hope these comments are constructive and encourage you to revise the manuscript for submission to a more suitable venue. The topic is valuable, and with improved theoretical framing, methodological clarity, and writing polish, this work could make a meaningful contribution to the digital transformation literature.
