# Supplementary material for: TBOE maturity assessment model for standard digitalization: An empirical analysis using AHP and Delphi method
Source: PLoS One. 2026 Mar 25;21(3):e0343406. doi: 10.1371/journal.pone.0343406 (PMC13016301; doi:10.1371/journal.pone.0343406)
Supplement: Attachment — Submitted filename: Reviewer_Comments_to_Editors.docx [file pone.0343406.s003.docx]

Dear Editors,

Thank you very much for the opportunity to review this manuscript. I’ve read the submission titled "TBOE Maturity Assessment Model for Digitalization of Standards" with interest. The authors present a maturity assessment model aimed at evaluating the digitalization of standards, using the electric power industry in China as a case study. The topic is relevant and timely, and the effort to construct a structured framework through AHP and Delphi methods is appreciated.

That said, after careful consideration, I find that the manuscript is not yet suitable for publication in this journal. Below, I outline the main reasons for this assessment.

**1. Theoretical Contribution Needs Clarification**
While the paper extends the classic TOE framework by incorporating a business dimension to form the TBOE model, the novelty of this extension is not sufficiently justified. As it stands, the model appears to be a reconfiguration of existing constructs without a clearly articulated theoretical advancement. For a submission to this journal, a stronger conceptual rationale would be needed.

**2. Limited Generalizability**
The current version of the manuscript focuses entirely on the electric power industry. While this is a significant sector, the lack of discussion on how the proposed model could be adapted or tested in other industries limits its broader relevance. Readers of this journal generally expect insights that extend beyond a single case or context.

**3. Methodological Depth Is Somewhat Shallow**
Although the authors employ AHP and Delphi methods, key details are lacking—especially regarding the selection criteria, diversity, and qualifications of the expert panel. Furthermore, no additional validation techniques (e.g., sensitivity analysis or scenario testing) are provided to reinforce the robustness of the model. As a result, the study feels more like a structured framework proposal rather than a fully realized empirical analysis.

**4. Writing and Structure Require Improvement**
The manuscript would benefit from a thorough language review. Many sections—including the abstract and introduction—feel overly long and include vague or awkward phrases (e.g., "digital standard is the linchpin of digital China"). Some terminology may not translate well for an international audience. Overall, the writing style detracts from the clarity and impact of the paper’s message.

**5. Insufficient Appeal for an International Readership**
The narrative is heavily anchored in the context of Chinese national policy. While these references are important for background, they dominate much of the discussion and may limit accessibility for international readers. A more neutral and globally-oriented framing would help improve the paper’s fit for this journal.

**Overall Recommendation**
In summary, although the authors have identified an important problem and made commendable efforts to address it, the manuscript in its current form falls short of the standards expected for publication in this journal. I recommend a substantial revision that includes stronger theoretical grounding, clearer methodological transparency, broader industry applicability, and professional English editing.

Thank you again for the opportunity to review this work.

Sincerely,
Sanggook Kim
