# Supplementary material for: TBOE maturity assessment model for standard digitalization: An empirical analysis using AHP and Delphi method
Source: PLoS One. 2026 Mar 25;21(3):e0343406. doi: 10.1371/journal.pone.0343406 (PMC13016301; doi:10.1371/journal.pone.0343406)
Supplement: Attachment — Submitted filename: Response to Reviewers_0827.docx [file pone.0343406.s006.docx]

Dear Reviewers:

Thank you very much for your suggestions on the paper. The following are the responses to each point.We hope the revised manuscript now meets the journal's high standards.

**Reviewer #1:**

1- The authors state that they integrated the TOE and DTF frameworks to construct the TBOE model; however, they do not provide sufficient explanation of these original frameworks. The manuscript should include a clear description of both TOE and DTF, along with a discussion of the existing literature that justifies the need for TBOE as an extension and highlights its added value.

**Response：**

At the beginning of “Mechanism of Action”, the sufficient explanation of the two original frameworks, TOE (Technology-Organization-Environment) and DTF (Digital Transformation Framework), were supplemented. Furthermore, the conceptual gaps of the two frameworks were analyzed, and the rationality and necessity of integrating the TOE framework and the DTF model, introducing the "Business" aspects of the DTF model into the TOE framework, and constructing the TBOE framework were proposed.

Specifically as follows:

TOE (Technology-Organization-Environment) framework is a comprehensive analysis framework based on the application scenarios of Technology and is widely used in the analysis of influencing factors in the process of new technology application and organizational transformation. Among them, the influence at the technical level mainly refers to the internal connection between technical characteristics and organizational structure. The influence at the organizational level mainly refers to the interaction between factors such as organizational structure and organizational management and the introduction of innovative technologies. The influence at the environmental level mainly refers to external conditions such as policy environment and industrial environment and the ecological environment.

DTF (Digital Transformation Framework) framework is a strategic evaluation model for digital transformation proposed by MattC et al. in 2015. It is applicable to the analysis of digital transformation in multiple fields under the current technological environment and consists of four elements. The first is the application of technology, which refers to the use of new technologies and the realization of their strategic goals. The second is value creation, which refers to the value impact of digital transformation on an organization. The third is the change in business structure, which refers to the transformation of business models caused by the realization of new technologies. The fourth is financial support, which refers to the financial conditions for applying new technologies to carry out digital transformation. It is the driving force for promoting changes in the first three aspects.

Both of the above frameworks emphasize the impact of technology on digital transformation. The TOE framework focuses on the role of factors such as organizational management in digital transformation, while the DTF model pays attention to the changes in business models and organizational structures triggered during the digital transformation process. The financial support factors proposed in the DTF model are one aspect of the organizational-level influencing factors in the TOE framework. The TOE framework has the inclusiveness to accommodate multiple theories and be applicable to various scenarios in analyzing the influencing factors of new technology applications, but it needs to enhance targeted interpretations for specific business domains. The DTF model analyzes the particularity of digital technology, focusing on the structural changes and value creation brought about by the application of digital technology. However, it is necessary to strengthen the analysis of organizational changes within the field.

Therefore, in accordance with the characteristics of digitalization of standards, this paper integrates the TOE framework and the DTF model, introduces the business factors of the DTF model into the TOE framework, expands the scope of the TOE framework, and constructs the TBOE analysis framework from four aspects: technical (T), business (B), organizational (O), and environmental (E).

2- The electric power industry is a compelling case study; however, the authors should discuss the model’s applicability to other industries and provide suggestions for future validation across different sectors.

**Response：**

The TBOE model developed in this study is a universal theoretical framework applicable to various industries or enterprises for assessing standard digitalization capability maturity, determining capability levels, and thereby informing subsequent path planning. However, when applying the model across different industries, the specific measurement indicators and scoring bases will vary. It is necessary to design tailored measurement indicators and scoring bases under the secondary indicators proposed herein, based on the unique characteristics of each industry. Among these, the indicators for the technical, organizational, and environmental factors can generally be reused directly. In contrast, the indicators under business factors will differ significantly across industries and must be designed based on the specific context of the industry itself.

This viewpoint has also been supplemented and elaborated in the paper.

3- Methodologically, the use of AHP and the Delphi method is appropriate. However, the authors need to provide more details regarding the selection criteria for experts, their backgrounds, and the full questionnaire used. The authors should include the full questionnaire as an appendix or supplementary file.

**Response：**

An expert advisory panel was formed by inviting specialists from the fields of standardization, digitization, and the power industry. Questionnaires were distributed through both online and offline channels. Experts were selected through three primary channels: 1) Standardization experts: Questionnaires were distributed through the Science and Technology Management Department to all researchers at the China National Institute of Standardization; 2) Digitalization experts: Targeted distribution was conducted to 85 member units of the National Standardization Working Group (SAC/SWG 29) and 10 project units under the National Key R&D Program "Key Technologies and Standards for Digital Evolution of Standards (Phase I)"; 3) Power industry experts: Questionnaires were distributed to relevant entities including the project team of State Grid Corporation's "Research on Implementation Pathways and Key Technologies for Standardization Digitalization", State Grid Information & Telecommunication Industry Group Co., Ltd., and China Electric Power Research Institute. The full questionnaire is shown in the Supporting information(S1)

Ultimately, 84 valid expert questionnaires were collected. The average age of the experts was 46.42 years, and 60.71% held senior professional titles. The experts were affiliated with institutions including China National Institute of Standardization, China Electronics Technology Standardization Institute, Zhejiang University, Zhijiang Laboratory, Shandong Provincial Computing Center (National Supercomputing Center in Jinan), State Grid Information and Communication Industry Group Co., LTD., China Electric Power Research Institute, State Grid Zhejiang Electric Power Co., LTD., Xi 'an Jiaotong University and other institutions.

4- The authors do not provide sufficient explanation of how the Delphi method was applied in this study. A brief overview of the Delphi method should be included, along with details such as the number of rounds conducted, how consensus was measured, and how expert feedback was processed.

**Response：**

The paper uses AHP to put forward the TBOE maturity assessment model for the digitalization of standards. The Delphi method is used in the process of index weight determination, and the Likert scale method is used in the process of index assignment.

In the "Methodology" chapter, the steps of the Delphi method are described at the theoretical level. In the chapter "Empirical Research and Model Verification", the application of the Delphi method is further supplemented.

5- The figures in the manuscript should be drawn again with clearer labels and improved resolution to enhance presentation quality.

**Response：**

The figures in the manuscript have been modified and the resolution has been improved.

**Reviewer #2:**

**1. Theoretical Justification and Model Positioning**
While the extension from TOE to TBOE is logical, the manuscript lacks a strong theoretical rationale for adding the "Business" component. Please clarify what conceptual gaps in the existing TOE or DTF frameworks your model fills. Including a comparative discussion of related maturity models or digital transformation theories could significantly improve the paper’s contribution.

**Response：**

At the beginning of the chapter on Mechanism of Action, the theoretical connotations of the two original frameworks, TOE (Technology-Organization-Environment) and DTF (Digital Transformation Framework), were supplemented. Furthermore, the conceptual gaps of the two frameworks were analyzed, and the rationality and necessity of integrating the TOE framework and the DTF model, introducing the "Business" aspects of the DTF model into the TOE framework, and constructing the TBOE framework were proposed. Specifically as follows:

TOE (Technology-Organization-Environment) framework is a comprehensive analysis framework based on the application scenarios of Technology and is widely used in the analysis of influencing factors in the process of new technology application and organizational transformation. Among them, the influence at the technical level mainly refers to the internal connection between technical characteristics and organizational structure. The influence at the organizational level mainly refers to the interaction between factors such as organizational structure and organizational management and the introduction of innovative technologies. The influence at the environmental level mainly refers to external conditions such as policy environment and industrial environment and the ecological environment.

DTF (Digital Transformation Framework) framework is a strategic evaluation model for digital transformation proposed by MattC et al. in 2015. It is applicable to the analysis of digital transformation in multiple fields under the current technological environment and consists of four elements. The first is the application of technology, which refers to the use of new technologies and the realization of their strategic goals. The second is value creation, which refers to the value impact of digital transformation on an organization. The third is the change in business structure, which refers to the transformation of business models caused by the realization of new technologies. The fourth is financial support, which refers to the financial conditions for applying new technologies to carry out digital transformation. It is the driving force for promoting changes in the first three aspects.

Both of the above frameworks emphasize the impact of technology on digital transformation. The TOE framework focuses on the role of factors such as organizational management in digital transformation, while the DTF model pays attention to the changes in business models and organizational structures triggered during the digital transformation process. The financial support factors proposed in the DTF model are one aspect of the organizational-level influencing factors in the TOE framework. The TOE framework has the inclusiveness to accommodate multiple theories and be applicable to various scenarios in analyzing the influencing factors of new technology applications, but it needs to enhance targeted interpretations for specific business domains. The DTF model analyzes the particularity of digital technology, focusing on the structural changes and value creation brought about by the application of digital technology. However, it is necessary to strengthen the analysis of organizational changes within the field.

Therefore, in accordance with the characteristics of digitalization of standards, this paper integrates the TOE framework and the DTF model, introduces the business factors of the DTF model into the TOE framework, expands the scope of the TOE framework, and constructs the TBOE analysis framework from four aspects: technical (T), business (B), organizational (O), and environmental (E).

**2. Generalizability and Broader Relevance**
Currently, the model is validated only within the Chinese electric power industry. While appropriate as a starting point, readers would benefit from discussion about how the model could apply across other sectors or geographies. Addressing generalizability would improve both scholarly relevance and practical utility.

**Response：**

The TBOE model developed in this study is a universal theoretical framework applicable to various industries or enterprises for assessing standard digitalization capability maturity, determining capability levels, and thereby informing subsequent path planning. However, when applying the model across different industries, the specific measurement indicators and scoring bases will vary. It is necessary to design tailored measurement indicators and scoring bases under the secondary indicators proposed herein, based on the unique characteristics of each industry. Among these, the indicators for the technical, organizational, and environmental factors can generally be reused directly. In contrast, the indicators under business factors will differ significantly across industries and must be designed based on the specific context of the industry itself.

This viewpoint has also been supplemented and elaborated in the paper.

**3. Methodological Transparency**
The description of the expert survey process needs significant elaboration. For example:
- How were the 84 experts selected?
- What were their backgrounds, affiliations, and expertise levels?
- What steps were taken to minimize bias in the Delphi process?

Including this information would help readers assess the robustness of the model-building process.

**Response：**

An expert advisory panel was formed by inviting specialists from the fields of standardization, digitization, and the power industry. Questionnaires were distributed through both online and offline channels. Experts were selected through three primary channels: 1) Standardization experts: Questionnaires were distributed through the Science and Technology Management Department to all researchers at the China National Institute of Standardization; 2) Digitalization experts: Targeted distribution was conducted to 85 member units of the National Standardization Working Group (SAC/SWG 29) and 10 project units under the National Key R&D Program "Key Technologies and Standards for Digital Evolution of Standards (Phase I)"; 3) Power industry experts: Questionnaires were distributed to relevant entities including the project team of State Grid Corporation's "Research on Implementation Pathways and Key Technologies for Standardization Digitalization", State Grid Information & Telecommunication Industry Group Co., Ltd., and China Electric Power Research Institute. The full questionnaire is shown in Supporting information(S1).

Ultimately, 84 valid expert questionnaires were collected. The average age of the experts was 46.42 years, and 60.71% held senior professional titles. The experts were affiliated with institutions including China National Institute of Standardization, China Electronics Technology Standardization Institute, Zhejiang University, Zhijiang Laboratory, Shandong Provincial Computing Center (National Supercomputing Center in Jinan), State Grid Information and Communication Industry Group Co., LTD., China Electric Power Research Institute, State Grid Zhejiang Electric Power Co., LTD., Xi 'an Jiaotong University and other institutions.

**4. Validation Depth**
The model relies heavily on AHP-derived consistency ratios (CR) as the primary form of validation. Consider adding additional checks, such as sensitivity analysis, to better establish confidence in the results. Even a limited robustness test could help improve the perceived reliability of your framework.

**Response：**

This paper validates the model through two approaches. The theoretical validation employs Analytic Hierarchy Process (AHP) consistency checks, including hierarchical single-ordering consistency tests and hierarchical total-ordering consistency tests, to verify the logical coherence of the judgment matrix. The applied validation conducts empirical research using the power industry as a case study. The model has been implemented at State Grid Corporation of China (SGCC), supporting its publication of the "Standard Digitalization White Paper". The derived indicator weight rankings from this model provide foundational evidence for SGCC's innovative standard digitalization models and pathway planning, thereby validating the model's authenticity and practical applicability at the operational level.

**5. Language and Writing Quality**
There are multiple instances of unclear or overly abstract phrasing that reduce readability. For example, the phrase “digital standard is the linchpin of digital China” may not be meaningful to readers outside the specific policy context. A thorough professional English review is strongly recommended to improve the tone, clarity, and precision of the text.

**Response：**

We have carefully addressed the concern regarding language quality by undertaking a thorough professional English editing of the entire manuscript. This process has significantly improved the clarity, tone, and precision of the text, enhancing its overall readability.

**6. Abstract and Structure**
The abstract is too lengthy and includes excessive methodological detail. Try to more concisely highlight the purpose, approach, and key findings of your study.

**Response：**

The abstract in the manuscript has been revised and simplified.

**7. Figures and Tables**
Some visuals can be improved:
- Figure 1 lacks clear definitions of directional arrows like “drive” and “push.”
- Figure 2 is dense and could be split into parts for easier interpretation.
- Table 4 is well-organized—consider visualizing it using bar charts to enhance accessibility.

**Response：**

The figures in the manuscript have been modified and the resolution has been improved, and table 4 is converted to a bar chart.

**8. References**
Make sure government and policy-related references are properly formatted and accessible to an international audience. When citing Chinese-language materials, translated titles and English summaries would be helpful.

**Response：**

The format and content of references in the manuscript have been calibrated.
