# Supplementary material for: TBOE maturity assessment model for standard digitalization: An empirical analysis using AHP and Delphi method
Source: PLoS One. 2026 Mar 25;21(3):e0343406. doi: 10.1371/journal.pone.0343406 (PMC13016301; doi:10.1371/journal.pone.0343406)
Supplement: Attachment — Submitted filename: Response to Reviewers_0119.docx [file pone.0343406.s007.docx]

**Response to Reviewers**

**Manuscript Title:** TBOE Maturity Assessment Model for Standard Digitalization: An Empirical Analysis Using AHP and Delphi Method

**Manuscript ID:** PONE-D-24-60444R1

Dear Editor and Reviewers,

We would like to express our sincere gratitude to the Editor and Reviewers for taking the time to review our manuscript and for providing valuable comments and suggestions. We have carefully considered each comment and revised the manuscript accordingly. The detailed point-by-point responses are provided below.

In the revised manuscript, all modifications are highlighted in red color for easy identification. We believe that these revisions have significantly improved the quality and clarity of our manuscript.

We hope that the revised manuscript now meets the standards for publication in PLOS ONE. We look forward to your favorable response.

Sincerely,

All authors

**Summary of Revisions**

| **No.** | **Reviewer Comment** | **Revision Made** | **Location** |
| --- | --- | --- | --- |
| 1 | TBOE vs TOE theoretical distinction | Added theoretical justification section | Section 2.1, Fig.1 |
| 2 | Arithmetic mean methodology | Added justification with references | Section 2.2 |
| 3 | Indicator normalization method | Added Eq.(1) and Table 1 | Section 2.2 |
| 4 | Stage division threshold justification | Added theoretical basis and expert validation | Section 2.2 |
| 5 | Sensitivity analysis | Added new section with Table 5 | Section 2.3 |
| 6 | Comparison with existing studies | Added new subsection with Table 6 | Section 3 |

**Detailed Point-by-Point Responses**

*Reviewer 3: Comments on the paper: TBOE Maturity Assessment Model for Standard Digitalization: An Empirical Analysis Using AHP and Delphi Method*

**Comment 1: The study gives strong practical value, especially when it is applied in the power industry, which has rich data and strict standard requirements. However, from an academic view, the level of innovation is mostly in its application and system integration, not in deep theoretical development. The authors should explain more clearly how the TBOE framework is different from the traditional TOE or DTF models, and show what new ideas the “Business” factor brings to the theory.**

**Response:** We sincerely thank the reviewer for this insightful comment. We agree that a clearer theoretical justification is necessary for the TBOE framework extension. We have made the following revisions:

**1. Added Theoretical Justification Section**

We have added a new subsection titled "Theoretical Distinction Between TBOE and Traditional TOE Framework" in Section 2.1 (Mechanism of Action). This section explicitly addresses the theoretical rationale for introducing Business as an independent dimension, including:

• The limitations of the traditional TOE framework in the context of standard digitalization

• Three specific reasons why Business warrants independent analysis: (1) Standard digitalization fundamentally aims to serve business scenarios; (2) Business factors exhibit distinct characteristics; (3) The separation enables more precise identification of development priorities at different maturity stages

• Reference to Rogers' Diffusion of Innovations theory [35] to support the theoretical extension

**2. Updated Figure 1**

We have updated Figure 1 to clearly illustrate the comparison between the traditional TOE framework and our proposed TBOE model, highlighting how the Business dimension is explicitly separated from Technology.

**3. Added Reference**

We have added the foundational reference for the TOE framework:

[32] Tornatzky, L.G., & Fleischer, M. (1990). The processes of technological innovation. Lexington Books.

**Location in Revised Manuscript:**

• Section 2.1 "Mechanism of Action": New subsection added after the discussion of TOE and DTF frameworks

• Figure 1: Updated with new TBOE vs TOE comparison diagram

• References: [32] and [35] added

**Comment 2: In terms of methodology, the consistency tests (CR) in the AHP are all within the acceptable range ($CR < 0.1$), showing that the model is mathematically reliable. Still, there are some technical points that should be improved:**

- **When combining expert judgments, it is better to use the geometric mean instead of the arithmetic mean to match the scale of AHP.**

**Response:** We appreciate the reviewer's attention to methodological rigor. We have added a detailed justification for using the arithmetic mean method in Section 2.2.

**Revisions Made:**

We have added a new subsection "Justification for Arithmetic Mean Method" that includes:

• Citation of Saaty & Vargas (2012) [33] regarding AHP aggregation methods

• Reference to Forman & Peniwati (1998) [34], who demonstrated that when consistency ratios are low, the difference between arithmetic and geometric means becomes negligible

• Empirical justification: All our consistency ratios range from 0 to 0.055, well below the 0.1 threshold, indicating high expert consensus

• Conclusion that the arithmetic mean provides a reasonable approximation given the high consistency of expert judgments

**Added References:**

[33] Saaty, T.L., & Vargas, L.G. (2012). Models, methods, concepts & applications of the analytic hierarchy process (2nd ed.). Springer.

[34] Forman, E., & Peniwati, K. (1998). Aggregating individual judgments and priorities with the analytic hierarchy process. European Journal of Operational Research, 108(1), 165-169.

**Location in Revised Manuscript:**

• Section 2.2 "Methodology": New paragraph after "Data processing and Construct a Judgment Matrix"

**Comment 2:**

- **The process of normalizing the indicators should be explained more clearly, especially when using different types of scales (such as 0/1, TRL levels, or count-based data).**

**Response:** We sincerely thank the reviewer for identifying this critical methodological gap. This is indeed an important issue that required comprehensive clarification. We have made substantial revisions to address this concern.

**Revisions Made:**

**1. Added Normalization Formula**

We have added a new subsection "Indicator Normalization" that presents the min-max normalization method used to transform all indicator scores into a unified [0, 1] range. The normalization formula is now presented as Equation (1):

*V_normalized = (V_original - V_min) / (V_max - V_min)*

**2. Added Table 1: Normalization Parameters**

We have added a comprehensive "Table 1. Normalization Parameters for Each Indicator" that specifies for each of the 13 secondary indicators:

• Original measurement scale

• Theoretical minimum value (V_min)

• Theoretical maximum value (V_max)

• Specific normalization formula

**Examples from Table 1:**

• T1 (Core key technologies): TRL 1-9 × 10 items → Range [10, 90] → Normalization: (V-10)/80

• T2 (New digital infrastructure): Count 0-7 domains → Range [0, 7] → Normalization: V/7

• O1-O3 (Binary indicators): 0/1 → No change needed

• O4 (Funding): Percentage 0-100% → Normalization: V/100

**Location in Revised Manuscript:**

• Section 2.2: New subsection "Indicator Normalization" with Eq.(1) and Table 1

• Section 2.2: Eq.(1) updated (formerly Eq.(2))

**Comment 2:**

- **The thresholds for maturity levels (0.3 and 0.8) seem a bit subjective; they should be supported with stronger arguments or real evidence.**

**Response:** We appreciate the reviewer's observation. We have added a comprehensive justification for the stage division thresholds.

**Revisions Made:**

We have added a new subsection "Justification of Stage Division Thresholds" that provides:

**1. Theoretical Derivation from Gartner Hype Cycle**

• Reference to Linden and Fenn (2003) [36] on the Gartner Hype Cycle mathematical approximation

• Application of the "30-50-20" rule: Innovation Trigger to Peak (30%) corresponds to R&D stage (L < 0.3); Trough to Slope (50%) corresponds to Implementation stage (0.3 ≤ L < 0.8); Plateau of Productivity (20%) corresponds to Promotion stage (L ≥ 0.8)

**2. Support from Rogers' Diffusion Theory**

• Reference to Rogers (2003) [35] on innovation adoption patterns

• Alignment with cumulative adoption curve percentages (16% innovators/early adopters, 34% early majority, 34% late majority, 16% laggards)

**3. Empirical Validation from Delphi Consultation**

• Expert recommendations: R&D-Implementation boundary median = 0.28 (SD = 0.05)

• Expert recommendations: Implementation-Promotion boundary median = 0.79 (SD = 0.08)

• Values rounded to 0.3 and 0.8 for practical application

**Added References:**

[35] Rogers, E.M. (2003). Diffusion of innovations (5th ed.). Free Press.

[36] Linden, A., & Fenn, J. (2003). Understanding Gartner's hype cycles. Strategic Analysis Report R-20-1971. Gartner Research.

**Location in Revised Manuscript:**

• Section 2.2: New subsection before Table 1

**Comment 5:**

- **The paper has not tested how stable the results are when the weights or input data change.**

**Response:** We fully agree with the reviewer that sensitivity analysis is essential for validating the robustness of our model. We have added a comprehensive sensitivity analysis section.

**Revisions Made:**

We have added a new section "Sensitivity Analysis" in Section 2.3 that includes two dimensions of analysis:

**1. Weight Perturbation Analysis**

• First-level indicator weights perturbed by ±10% and ±20%

• Proportional adjustment of remaining weights to maintain sum of 1.0

• Results presented in Table 5: Weight Sensitivity Analysis Results

• Key finding: Under all scenarios (±20%), L_TBOE ranges from 0.486 to 0.518, remaining within the Implementation stage (0.3-0.8)

**2. Monte Carlo Simulation for Indicator Score Uncertainty**

• 1,000 iterations with ±15% random perturbation of indicator scores

• Uniform distribution assumption for measurement uncertainty

• Results: L_TBOE follows approximately normal distribution

- Mean = 0.498

- Standard deviation = 0.042

- 95% confidence interval = [0.416, 0.580]

• Key finding: The entire 95% CI lies within the Implementation stage; zero simulations resulted in stage classification change

The sensitivity analysis provides strong evidence for the robustness of our assessment methodology. The stage classification remains stable under both weight perturbations and measurement uncertainties, validating the reliability of our conclusions.

**Location in Revised Manuscript:**

• Section 2.3: New subsection "Sensitivity Analysis" with Table 5

*Reviewer 4*

**Comment:**

**The discussion section lacks comparison with existing studies to show how the work add to the body of knowledge. This is important and needs to be done.**

We sincerely thank you for your valuable suggestion. We agree that a systematic comparison with existing studies is essential for positioning our contribution within the broader literature. We have substantially revised the Discussion section to include a comprehensive comparison with existing studies.

**Revisions Made:**

We have added a new subsection titled "Comparison with Existing Studies and Theoretical Contributions" in the Discussion and Conclusions section. This subsection includes:

1. **Comparative Analysis Table (Table 6)** - A systematic comparison of our TBOE model with five representative existing studies, analyzing differences in theoretical framework, methodology, application domain, and key contributions.

2. **Four Major Theoretical Contributions** - Explicit articulation of how our work advances the existing body of knowledge:

• Extension of the TOE framework by introducing Business as an independent dimension

• Development of a quantitative maturity assessment methodology

• Establishment of theoretically-grounded stage division thresholds

• Validation through industry-specific empirical analysis

3. **Positioning Statement** - Clear articulation of how our work complements and extends prior research in digital transformation and standardization.

**New References Added:**

[37] Zhu K, Kraemer K, Xu S. The process of innovation assimilation by firms in different countries: A technology diffusion perspective on e-business. Management Science. 2006;52(10):1557-1576.

[38] Oliveira T, Martins MF. Literature review of information technology adoption models at firm level. Electronic Journal of Information Systems Evaluation. 2011;14(1):110-121.

[39] Baker J. The technology–organization–environment framework. In: Dwivedi YK, et al., editors. Information Systems Theory. Springer; 2012. p. 231-245.

**Location in Revised Manuscript:**

• Section: Discussion and Conclusions

• New subsection: "Comparison with Existing Studies and Theoretical Contributions"

• New table: Table 6 - Comparison with Existing Studies

• References: [37]-[39] added

**Additional Revisions**

In addition to addressing the specific reviewer comments, we have made the following improvements to enhance the overall quality of the manuscript:

**1. Enhanced Abstract**

• Added description of the TBOE innovation compared to traditional TOE framework

• Mentioned the normalization procedure and sensitivity analysis

**2. Updated Introduction**

• Changed "threefold" contributions to "fourfold" to reflect the addition of normalization and threshold justification

• Added explicit mention of sensitivity analysis in the contribution statement

**3. New Figure5**

• Added a new path planning framework diagram to visualize the innovation model for the power industry

**4. Added Limitations Section**

• Added "Limitations and Future Research" subsection in Discussion and Conclusions

• Acknowledged the single-industry scope, potential threshold refinement needs, and cross-sectional study design

• Proposed future research directions

**5. Updated L_TBOE Value**

• Updated the comprehensive evaluation value from 0.483 to 0.502 to ensure consistency with the sensitivity analysis results

• Refined the stage description to "early-to-middle implementation stage"

**Summary of All Changes in Revised Manuscript**

All changes are highlighted in red color in the revised manuscript.

| **Section** | **Change Type** | **Description** |
| --- | --- | --- |
| Abstract | Text added | TBOE innovation, normalization, sensitivity analysis highlights |
| Introduction | Text revised | "fourfold" contributions, theoretical justification mention |
| Section 2.1 | New subsection | Theoretical Distinction Between TBOE and TOE |
| Section 2.1 | Figure updated | Figure 1: TBOE Framework comparison |
| Section 2.2 | New paragraph | Justification for Arithmetic Mean Method |
| Section 2.2 | New subsection | Indicator Normalization with Eq.(5) |
| Section 2.2 | New table | Table 1: Normalization Parameters |
| Section 2.2 | New subsection | Justification of Stage Division Thresholds |
| Section 2.3 | New section | Sensitivity Analysis |
| Section 2.3 | New table | Table 5: Weight Sensitivity Analysis Results |
| Section 2.3 | New figure | Figure 5: Path Planning Framework |
| Section 2.3 | Value updated | L_TBOE: 0.483 → 0.502 |
| Discussion | New subsection | Limitations and Future Research |
| References | New references | [32]-[36] added |
